# Linking DNA Damage and Age-Related Promoter DNA Hyper-Methylation in the Intestine

**DOI:** 10.3390/genes9010017

**Published:** 2018-01-05

**Authors:** Torsten Thalheim, Maria Herberg, Joerg Galle

**Affiliations:** Interdisciplinary Centre for Bioinformatics, Leipzig University, Haertelstr. 16-18, 04107 Leipzig, Germany; herberg@izbi.uni-leipzig.de (M.H.); galle@izbi.uni-leipzig.de (J.G.)

**Keywords:** intestinal stem cell aging, computational modeling, promoter hyper-methylation, DNA damage repair, epigenetic states

## Abstract

Aberrant DNA methylation in stem cells is a hallmark of aging and tumor development. Here, we explore whether and how DNA damage repair might impact on these time-dependent changes, in particular in proliferative intestinal stem cells. We introduce a 3D multiscale computer model of intestinal crypts enabling simulation of aberrant DNA and histone methylation of gene promoters during aging. We assume histone state-dependent activity of de novo DNA methyltransferases (DNMTs) and methylation-dependent binding of maintenance DNMTs to CpGs. We simulate aging with and without repeated DNA repair. Motivated by recent findings on the histone demethylase KDM2b, we consider that DNA repair is associated with chromatin opening and improved recruitment of de novo DNMTs. Our results suggest that methylation-dependent binding of maintenance DNMTs to CpGs, establishing bistable DNA methylation states, is a prerequisite to promoter hyper-methylation following DNA repair. With this, the transient increase in de novo DNMT activity during repair can induce switches from low to high methylation states. These states remain stable after repair, leading to an epigenetic drift. The switches are most frequent in genes with H3K27me3 modified promoters. Our model provides a mechanistic explanation on how even successful DNA repair might confer long term changes of the epigenome.

## 1. Introduction

In the last decade, genome-wide analysis of epigenetic marks provided evidence that aging of tissue stem cells is accompanied by changes in the epigenetic profiles of these cells. Probably, the best studied example is aberrant DNA methylation. In many tissues, a global hypo-methylation is observed with increasing age. In parallel, a tissue-specific set of gene promoters become hyper-methylated [1]. These changes are associated with the proliferation history of the cells [2]. They have been successfully used to predict the chronological age of individuals [3,4,5]. Moreover, accelerated DNA methylation changes have been linked to the emergence of several diseases [6,7]. In particular, accelerated promoter hyper-methylation has been highlighted as a hallmark of tumor development [8]. Thereby, DNA methylation changes strongly correlate with a specific histone modification pattern. Gene promoters associated with nucleosomes that show tri-methylation of lysine 27 at histone 3 (H3K27me3) are prone to hyper-methylation during aging and tumor formation [8,9]. In embryonic stem cells, the same promoters are frequently associated with nucleosomes that show tri-methylation of lysine 4 at histone 3 (H3K4me3) [10]. The respective bivalent genes are known to be enriched in developmental processes [11].

Approaching histone and DNA methylation dynamics from a theoretical side, we recently hypothesized that age-related changes of DNA methylation originate from a limited inheritance of histone methylation marks [12,13]. Thereby, strong dilution of histone marks in daughter cells following cell division opens a time window for the de novo DNA methyltransferases DNMT3a/b to become active at promoters that are otherwise protected against DNA methylation. Such a scenario can be expected to induce DNA hyper-methylation in slowly proliferating stem cells that become proliferative on demand, e.g., in case of tissue repair. In continuously proliferating tissues, corresponding DNA methylation changes develop on a short time scale of a few weeks, i.e., they do not proceed on a life-long time scale. Thus, they cannot be considered as age-related.

The intestinal epithelium is a fast, self-renewing tissue. In humans, it regenerates entirely within about one week. However, also in this highly proliferative tissue, hyper-methylation of gene promoters has been observed during aging and tumor formation in mice and humans [14,15]. Also here, hyper-methylation is found predominately at H3K27me3 marked promoters. Analyzing the stability of H3K27me3 in CpG-rich regions of the small intestine of mice, more than 3500 regions were found to decrease H3K27me3 levels following colitis [16]. These effects are most probably linked to increased DNA damage [17]. Age-related studies of H3K4me3 modification levels in the intestine are currently missing. Here, we aim at a mechanistic explanation of the origin of hyper-methylation in the intestine and the role of histone modifications in this process.

One potential explanation of age-related hyper-methylation in the intestine is frequent DNA damage repair (DDR). Intestinal stem cells (ISCs) are prone to proliferation-associated DNA damage because of their high proliferative activity. Efficient repair of the damage, in particular if localized at epigenetically repressed genes, requires local chromatin opening in order to enable access of the repair machinery [18]. Such changes ideally have to be, but are potentially not, completely reversible. We here hypothesize that promoter hyper-methylation in ISCs is driven, at least in part, by regulatory effects associated with DDR. Building on previous studies, we introduce a 3D multiscale computational model of the intestinal crypt and apply it to study the consequences of chromatin dynamics known to be associated with DDR. In the model, each cell contains a random genome. The encoded genes are regulated by a transcription factor (TF) network and by chromatin modifications associated with the gene’s promoters. The multiscale model allows for the study of individual crypt cells throughout their whole life time. Thus, it enables the analysis of age-related epigenetic changes of them depending on their crypt position, lineage type, and proliferation activity.

In the following, we first introduce our computational model. Subsequently, we provide simulation results on damage-free aging and aging under repeated, successful DDR. In particular, we focus on the long term epigenetic changes associated with repeated successful DDR.

## 2. Methods

### 2.1. 3D Multiscale Model of Epigenetic Aging of the Intestinal Epithelium

Our model builds on our previous studies on ISC self-organization in the crypt [19,20] and on age-related epigenetic drifts in stem cells [13]. It combines the 3D individual cell-based model of the intestinal crypt with the model of epigenetic regulation of transcription as described in those studies, respectively. Thereby, it considers all extensions of the model of epigenetic regulation introduced in [21].

#### 2.1.1. The Model of Epigenetic Regulation of Transcription 

**Figure 1:**
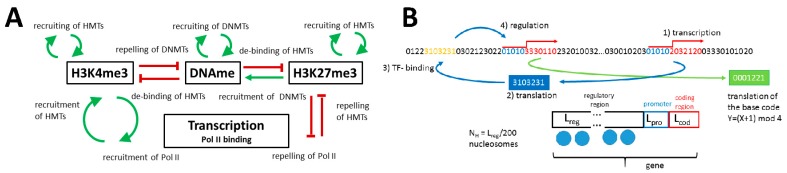
**Model of epigenetic regulation of transcription:** (**A**) Interactions between histone modifications, DNA methylation, and transcription considered in the model. (**B**) Random genome approach: A single-stranded genome (four types of bases) is composed of genes of fixed length. Each gene consists of a regulatory region (*L_reg_*), a promoter (*L_pro_*), and a coding sequence (*L_cod_*). The coding sequence is transcribed (1) and subsequently translated into a transcription factor (TF) (2). If a TF binds to matching sequences in the regulatory regions of other genes (3), these genes are transcriptionally regulated by the TF (4). In this way, a dynamic TF-network is generated. *L_reg_*/200 cooperative nucleosomes are assumed to be associated with the regulatory region of a particular gene and to contribute to its regulation. HMT: histone methyltransferase; DNMT: DNA methyltransferase.

The model of epigenetic regulation of transcription (T) considers histone methylation at H3 (H3K4me3, H3K27me3) and DNA methylation. It describes dynamic histone and DNA methylation states due to stochastic histone (de-)modification processes. The model quantifies the histone and DNA methylation levels as the fraction of modified nucleosomes (*m_4_*, *m_27_*) and of methylated CpGs (*m_DNA_*) associated with the promoter, respectively. Thereby, it applies to CpG-rich gene promoters. The histone modification processes are controlled by binding of the respective histone methyltransferase (HMT) complexes to nucleosomes and the associated DNA. This binding depends on the transcriptional activity of the associated genes and feeds back on their transcription via the established modifications. An overview of all molecular interactions considered in the model is provided in Figure 1A. We assume that transcriptional activity facilitates recruitment of H3K4me3 HMTs [22] and that H3K4me3 contributes in recruiting Pol II [23]. In parallel, transcriptional activity suppresses the recruitment of the H3K27me3 HMTs [24], and H3K27me3 impedes recruitment of Pol II [25]. During cell division, modified histones associated with a gene promoter of the mother cell are randomly distributed along the same promoter of the two daughters [26] and are complemented with unmodified ones. This results in a strong histone de-modification of the promoter. Depending on its stability, the modification state present in the mother cell can subsequently become reestablished or the promoter spontaneously de-modifies. Details of the implementation can be found in [21].

DNA methylation is controlled by a histone state-dependent activity of de novo DNA methyltransferases (DNMTs) and by the activity of maintenance DNMTs. Both types of DNMTs are assumed to be active only subsequent to cell division. We assume the following regulatory feedback loops between DNA methylation and histone modifications: H3K4me3 suppresses binding of de novo DNMTs [27] and DNA methylation weakens the binding of H3K4me3 HMTs to DNA [28]. In addition, DNA methylation also weakens binding of H3K27me3 HMTs [24], while H3K27me3 itself contributes in recruiting de novo DNMTs [29]. DNA methylation is assumed to have no direct impact on transcription in accordance with experimental findings showing that transcriptional silencing of promoters precedes their DNA methylation [30]. In extension to [21], we assume a DNA methylation-dependent binding of maintenance DNMTs to CpGs, controlling their activity. Details of the underlying binding model are provided in the Results section. Equations and parameters are given in Appendix A1 and Appendix A2.

In order to implement these interactions, a random genome approach [31] is applied (Figure 1B). The genes encoded by the random genome can be transcribed and afterwards translated into transcription factors (TFs). The TFs encode an entire network, which defines a first layer of transcriptional regulation of the genes. In contrast to [13], we assembled the genome from random genes of fixed length, to enable direct comparison to [21]. A second regulatory layer is defined by the epigenetic modifications of the nucleosomes associated with the regulatory region of the genes.

#### 2.1.2. The 3D Individual Cell-Based Model of the Intestinal Crypt

**Figure 2:**
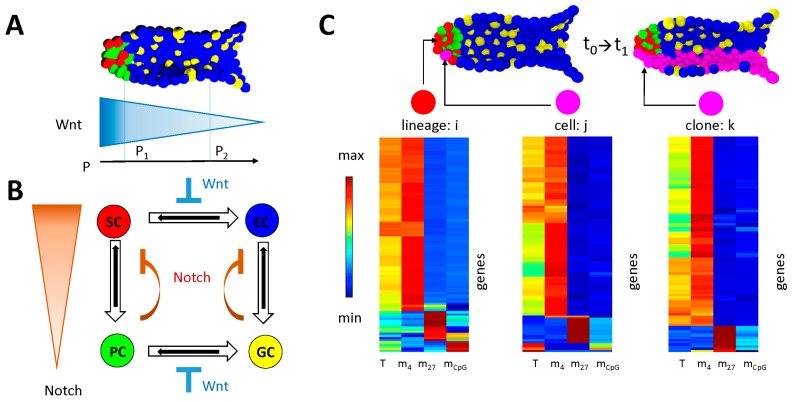
**Multiscale model of the crypt.** (**A**,**B**) Intestinal stem cell (ISC) maintenance requires both high Wnt- and high Notch-signaling. (**A**) Wnt-signaling is provided extrinsically by stroma cells and forms a gradient with the highest signal at the bottom of the crypt. (**B**) Notch-signaling is induced in ISCs and enterocytes (ECs) by secretory neighbor cells (Paneth cells (PCs) and Goblet cells (GCs)). (**C**) Each cell of the crypt incorporates a random genome that encodes 100 genes. The regulatory state of each cell, defined by the values of *T*, *m_4_*, *m_27_*, and *m_CpG_* of all these genes, is updated individually. The model allows for the analysis of the average cell state of a lineage (color coding see (**B**)) at each time point *t_0_*, but also the state of a selected cell (magenta) at this time point or the state of the clone arising from this cell at later times (*t_1_*).

The 3D individual cell-based model of the intestinal crypt enables simulations of stem cell organization and lineage specification in the small intestine of mice. It describes these processes based on the activity of Wnt- and Notch- signaling (Figure 2A,B). It allows specification of ISCs into enterocytes (ECs), Paneth cells (PCs), and Goblet cells (GCs). Details can be found in Appendix A3. 

In order to analyze epigenetic regulation in the intestinal crypt, we incorporated a random genome into each cell of the crypt model and applied the epigenetic model of transcriptional regulation to each gene encoded by the genome. Thereby, the expression of these genes does not affect the ISC-regulation that controls the lineage specification. We utilized the gene regulatory model to study long term epigenetic changes that refer to the proliferative activity of the cells and to repeated, successful DDR.

### 2.2. Model of DNA Damage and Damage Repair in the Crypt

Each DDR process is associated with chromatin opening to guarantee accessibility of the damaged DNA [18]. Related assumptions in our model are motivated by experimental observations associated with DNA double-strand breaks (DSBs) described in [32]. In particular, we assume that DNA damage induces fumarate, which represses the histone demethylases (HDM) KDM2b, known to be a strong H3K4me3 HDM [33]. This repression is modeled by decreasing the demethylation constant of H3K4me3 ($C_{de}^{4}$). Antagonistically, the maximum de novo DNA methylation probability (*D_novo,0_*) is increased for the time of repair. The latter considers the protective function of KDM2b for gene promoters regarding DNA methylation [34], which is lost with its repression.

DNA damage is assumed to occur randomly throughout the genome and with a constant probability per cell division for each gene of each cell in the crypt. In any case, the damage is assumed to be fully repaired by DDR during a fixed time within the cell cycle in which it occurs. For simplification, we assume no extension of the cell cycle according to ongoing DDR.

## 3. Results

### 3.1. Maintenance of DNA Methylation with Positive Auto-Feedback

Positive feedback mechanisms in DNA methylation processes have been suggested already by others [35,36] and are supported by experimental data [37]. Here, in extension to our model applied in former studies [12,13], we assume a particular strong recruitment of DNMT1 to methylated CpGs if surrounding CpGs are methylated as well. This assumption is supported by recent experimental findings proposing a cooperative action of the methyl-CpG-binding domain protein MBD4 and DNMT1 [38,39]. We assume that the DNMT1 binding probability to the promoter depends on the methylation level *m_CpG_* of the CpGs at this promoter:

(1) $$D_{main}=D_{main,0}/\left(1+\exp\left(E_{m,0}+E_{m,1}m_{CpG}\right)\right)$$

This binding is controlled by two energy constants *E_m,0_* and *E_m,1_* describing accessibility for and binding of DNMT1 to methylated CpGs of the promoter, respectively. According to this feedback, the DNA methylation becomes bistable for defined parameter sets. The range of bistability is controlled by the energy constants (*E_m,0_*, *E_m,1_*), the maximum probability of maintaining a methyl-CpG in the daughter (*D_main,0_*), and by the effective de novo DNA methylation probability *D_novo_*(*m_4_*, *m_27_*). The latter depends on the histone modification states of the gene under consideration [21]:

(2) $$D_{novo}=D_{novo,0}\left(1+\exp\left(-E_{CpG}^{27}m_{\mathrm{27}}\right)\right)/\left(1+\exp\left(E_{CpG}^{4}m_{4}-E_{CpG}^{27}m_{\mathrm{27}}\right)\right)$$

where $E_{CpG}^{4}$ and $E_{CpG}^{27}$ are energy constants modulating the binding of de novo DNMTs to the promoter in presence of the respective modification. In contrast to [35], *D_novo_* does not depend on the DNA methylation state. The changes Δ*m_CpG_* of the DNA methylation subsequent to cell division (to reach a balance between maintenance and de novo DNA methylation) are than described by:

(3) $$∆m_{CpG}=D_{novo}\left(m_{4},m_{\mathrm{27}}\right)\left(1-m_{CpG}\right)-\left(1-D_{main}\left(m_{CpG}\right)\right)m_{CpG}$$

**Figure 3:**
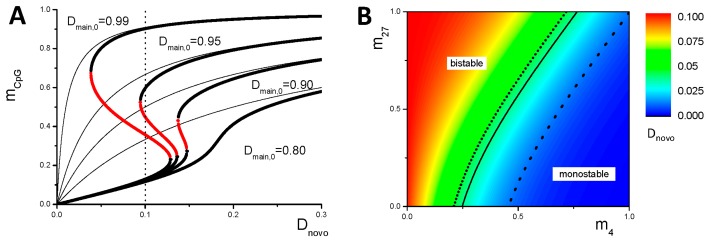
**DNA methylation-dependent binding of maintenance de novo DNA methyltransferases.** (**A**) Numerical solutions of *m_CpG_* (Equations (1)–(3)) in dependence of *D_novo_* for different values *D_main,0_*. Stable and unstable solutions are shown in black and red, respectively. Solid lines represent solutions used in [21] resulting in higher *m_CpG_* at low *D_novo_*. The vertical dotted line refers to the maximum value of *D_novo_* in our simulations without repair (*D_novo_*_,*0*_ = 0.1). (**B**) *D_novo_* in dependence of the histone modification states (*m_4_*, *m_27_*) for *D_novo,0_* = 0.1. The solid line separates regions of mono-stable (low *m_CpG_*) and bi-stable solutions of *m_CpG_* for *D_main,0_* = 0.99. Setting *D_novo,0_* = 0.3 during repair, the bi-stable region above the short-dotted line becomes mono-stable (high m_CpG_) and the region between the two dotted lines becomes bistable.

**Figure A1:**
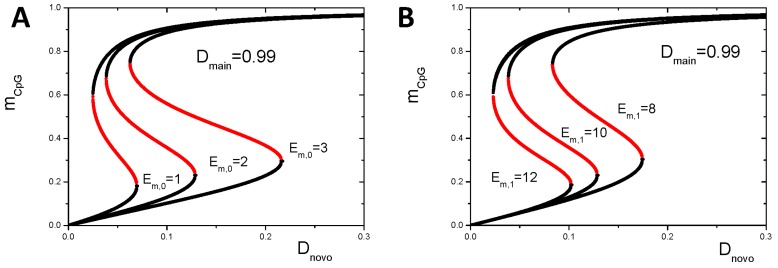
**DNA methylation- dependent binding of maintenance DNMTs.** Solutions of Equations (1)–(3) for *m_CpG_* assuming different parameter values of (**A**) *E_m,0_* (for *E_m,1_* = 10) and (**B**) *E_m,1_* (for *E_m,0_* = 2). Moderate changes of these parameters lead to similar systems behavior. Thereby, increasing *E_m,0_* simply extends the region of bistable solutions, while increasing E_m,1_ moves this region to smaller values of *D_novo_*.

Figure 3A shows the dependence of the equilibrium DNA methylation level m_CpG_ on the de novo DNA methylation probability *D_novo_*(*m_4_*, *m_27_*). For high *D_novo_*(*m_4_*, *m_27_*), the dependence of *m_CpG_* on *D_novo_*(*m_4_*, *m_27_*) is similar to that applied in our former studies. In contrast, it shows a hysteresis behavior at low values (in particular for high *D_main,0_*). The dependence of *D_novo_*(*m_4_*, *m_27_*) on the histone modification states m_4_ and m_27_ is illustrated in Figure 3B. For H3K4me3 modified (*m_4_* > 0.5, *m_27_* < 0.5) promoters, *D_novo_*(*m_4_*, *m_27_*) is very low. Under these conditions, only one stable DNA methylation state exists corresponding to low DNA methylation. Changing to bivalent (*m_4_*, *m_27_* > 0.5) or unmodified states (*m_4_*, *m_27_* < 0.5) increases *D_novo_*(*m_4_*, *m_27_*) and two stable DNA methylation states can exist. A H3K27me3 state (*m_4_* < 0.5, *m_27_* > 0.5) is related to an even higher *D_novo_*(*m_4_*, *m_27_*). Here, for high *D_novo_,_0_* (Figure 3B), again, a single DNA methylation state can occur, corresponding to a high DNA methylation level. In the following, we assume under intestinal homeostasis *D_novo,0_* = 0.1 (Figure 3A). Accordingly, mono-stable high-methylation states are not reached for the parameter set applied (Table A1). Solutions for different values of *E_m,0_* and *E_m,1_* are provided in Appendix B (Figure A1).

### 3.2. Simulation of Aging without Damage

We applied the multiscale model to simulate epigenetic aging in intestinal crypts of mice. Our crypts contained on average 9 (±4) ISCs, 32 (±3) PCs, 59 (±6) GCs and 222 (±10) ECs (compare: [19]). These numbers self-organize according to the assumed specification rules (Figure 2A). We ran simulations of 300 days of lifetime and followed the changes of the epigenome and of the transcriptome of all cells. In the first series of simulations, we simulated epigenetic aging without damage. We started with different initial conditions for *m_4_* and *m_27_*. In either case, we set *m_CpG_*(*t* = 0) = 0 for all genes. We analyzed the systems behavior for all four cell lineages of the crypt separately, because all of them have different proliferation dynamics. 

**Figure 4:**
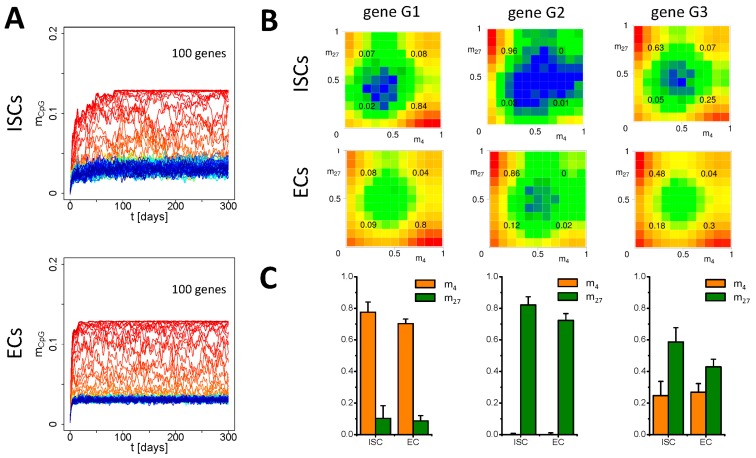
**Simulation of damage-free aging.** (**A**) DNA methylation level *m_CpG_* of all genes averaged over all cells of a given lineage (ISCs and ECs). Line colors are distributed according to the mean level of *m_CpG_* over time. Homeostasis is reached after about 6–8 weeks and 2–3 weeks for ISCs and ECs, respectively. (**B**) Simulated distribution of the modification states (*m_4_*, *m_27_*) for three different genes (G1: H3K4me3 target, G2: H3K27me3 target, G3: H3K4me3 and H3K27me3 target) as detected in ISCs and ECs. Colors indicate the logarithmic frequency of occurrence. (**C**) Averaged (*m_4_*, *m_27_*) states. The average was taken over all cells and subsequently over all time points *t* > 30 days. (Error: standard deviation (SD) of the population average).

**Figure A2:**
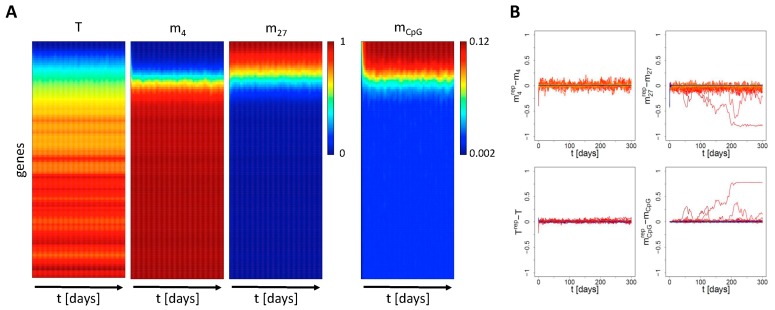
**Regulatory states during aging:** (**A**) Heat maps of the normalized logarithm of transcription (*T*) and of histone (*m_4_*, *m_27_*) and DNA methylation (*m_CpG_*) states for all genes during damage-free aging. The genes are clustered according to their *m_CpG_* values. (**B**) Effects of DDR. Comparison of the regulatory states of all genes for damage-free and repair-induced aging (Index: rep). The differences for logarithm of the transcription (*T*) and H3K4me3 levels (*m_4_*) are rather small for most of the genes. Genes that underwent hyper-methylation lose H3K27me3 (*m_27_*). Line color code as in Figure 4A.

We found that both the epigenome (Figure 4A: *m_CpG_*, Figure A2A: *m_4_*, *m_27_*) and the transcriptome (Figure A2A: *T*) become stable in all lineages after a few weeks (2–3 weeks in ECs, 6–8 weeks in ISCs) independent of the initial conditions for *m_4_* and *m_27_*. During this initial time, epigenetic drifts are observed as detected in simulations of slowly proliferating stem cells [13]. In the subsequent phase, we observed normal fluctuations of all properties but no further drifts, in particular no DNA methylation drifts (Figure 4A). Actually, not a single gene in any cell switched from low to high DNA methylation during this phase. 

Overall, we observed only small differences in histone modification states between the cell lineages. Figure 4B shows the histone state distributions for ISCs and ECs for three selected genes: one H3K4me3 target (G1), one H3K27me3 target (G2), and one gene that switches between these states (G3). In accordance with the results in [21], we found an increase in unmodified states at the expense of strongly modified states and of bivalent states as a consequence of progenitor cell proliferation (see: Figure 4B, inserted numbers). During specification of ISCs into ECs, the most pronounced effect is loss of H3K27me3, as e.g., shown for gene G3 (Figure 4C). Loss of H3K27me3 in crypts during lineage specification into ECs have been also observed experimentally [40]. 

### 3.3. Simulation of Aging under Repeated DDR

**Figure 5:**
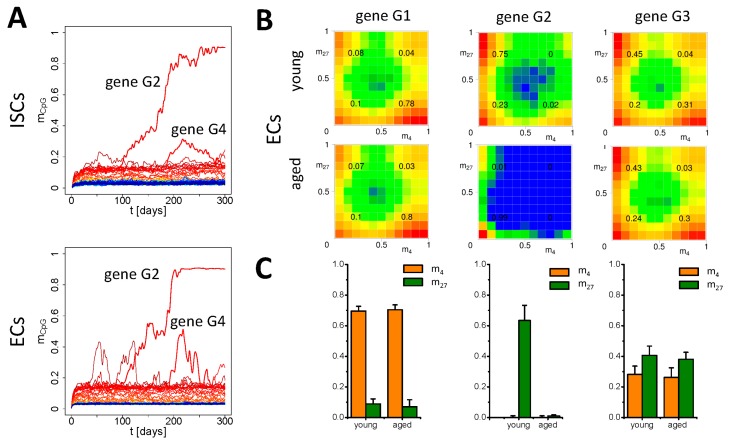
**Simulation of aging under DNA damage repair (DDR).** (**A**) DNA methylation level of all genes averaged over all cells for ISCs and ECs. One gene (G2) switches into a high-methylation state, while others (e.g., G4) increase methylation only transiently. Line color code as in Figure 4A. (**B**) Simulated modification states (*m_4_*, *m_27_*) for genes G1-3 (see Figure 4B) as detected in ECs of young (30–130 days old) and aged (200–300 days old) crypts. (**C**) Average (*m_4_*, *m_27_*) states. The average was taken over all cells and subsequently over the indicated time windows. (Error: sd of the population average).

**Figure A3:**
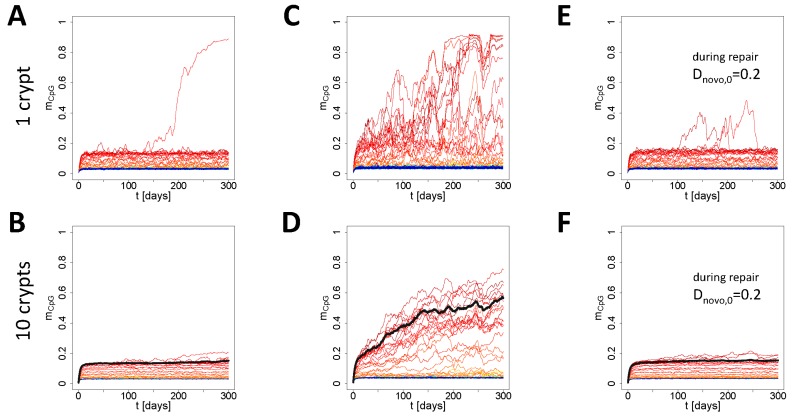
**DNA methylation drifts under low and high DDR frequencies**. (**A**) For one repair event per cell cycle and cell, single events of hyper-methylation fixation can be observed. (**B**) The switch-like behavior of *m_CpG_* averaging over all cells in one crypt, changes to drift-like behavior averaging of several crypts. The resulting drifts, however, are marginal. (**C**) For ten repair events per cell cycle and cell, on average, 5.6 hyper-methylation events become fixed within 300 days. (**D**) This results in strong drifts. (**E**) Decreasing *D_novo,0_* to *D_novo,0_* = 0.2, fixation of hyper-methylation is not detected even under the high repair frequency, and only temporarily changes of *m_CpG_* occur. (**F**) Averaging over several crypts, a slightly increased level of *m_CpG_* is observed for a set of genes but no drifts. Color code as in Figure 4A. The black lines in (**B**,**D**,**F**) indicate the average over the ten most methylated genes.

In a second series of simulations, we analyzed the effects of chromatin opening during DNA repair. Again, we simulated crypt behavior for 300 days and analyzed the time development of the transcriptome and the epigenome. Figure 5 and Figure A2B show selected simulation results assuming that two genes out of the 100 encoded by the genome are subject to repair per cell cycle and cell. Simulation results for other repair frequencies can be found in the Appendix B (Figure A3). For all simulations, we used the damage-free scenario as a control.

In contrast to the control scenario, we observed a general increase of the average DNA methylation. Figure 5A shows a simulation where the DNA methylation status of a single gene changes from low to high, (the H3K27me3 target gene G2, Figure 4B) while that of other genes (among them gene G4) increases only transiently. Thereby, the distribution of the histone states of most of the genes is only weakly disturbed by the repair (Figure A2B). Especially, the H3K4me3 modification level *m_4_* remains almost constant, except of the decreased H3K4me3 HDM activity during repair. However, the histones of the few genes that become hyper-methylated completely de-modify. This is shown in Figure 5B. Here, the histone state distribution in young and aged cells is compared for the same genes as selected for Figure 4B. The H3K27me3 target gene G2 completely de-modifies in the course of hyper-methylation, while G1 and G3 remain unaffected. Figure 5C compares the average modification states of the genes in the given time windows.

Transitions between the methylation states occur on a relatively short time scale, i.e., during a few cell cycles. A switch-like behavior on a time scale of 6–8 weeks is still observed when averaging m_CpG_ over all cells of a selected lineage (Figure 5A). This refers to the time scale of monoclonal conversion in the crypts [41]. Transitions are observed for a maximum de novo methylation probability during repair of *D_novo,0_* = 0.3. By decreasing this value to 0.2, genes only transiently change their methylation state. This effect cannot be compensated even by a 5-fold higher repair frequency (Figure A3E,F).

**Figure 6:**
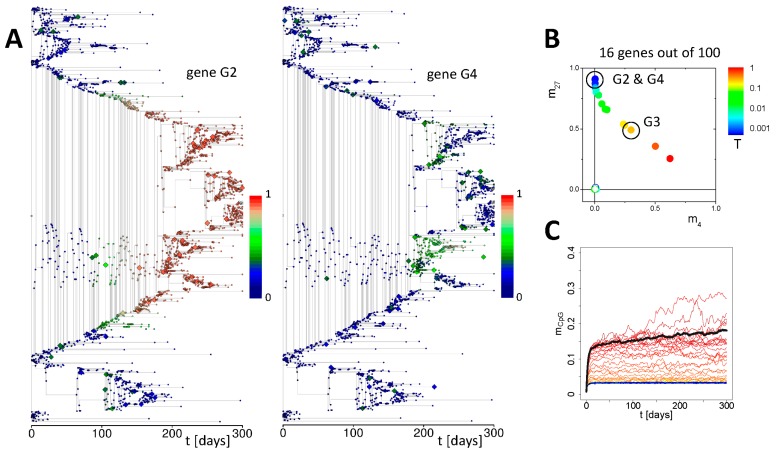
**Properties of hyper-methylated genes.** (**A**) Example of a phylogenetic tree calculated for all ISCs and progenitor cells based on the simulation underlying the results presented in Figure 5. The colors indicate the methylation level of gene G2 (left) and gene G4 (right) that underwent hyper-methylation with and without fixation in the crypt, respectively (compare: Figure 5A). Large symbols indicate repair events in a particular cell. (**B**) Control histone modification states of genes that underwent hyper-methylation in at least one of our simulations (solid symbols, all repair frequencies). Open symbols indicate their modification states subsequent to hyper-methylation. The color code indicates the transcription level. Most genes are H3K27me3 targets in controls but in a few cases carry also H3K4me3. (**C**) DNA methylation of all genes averaged over all cells and over ten different crypts. Line color code as in Figure 4A. The black line indicates the average over the ten most methylated genes.

We used phylogenetic trees to visualize the simulated ISC and progenitor history [19,42]. In these trees the edges represent cells and each vertex represents a mitotic event. The length of an edge is defined by the individual cell cycle time. After mitosis, the outgoing edges indicate the daughters arising from the mother. In case the edges are orientated vertically, both daughters remain undifferentiated otherwise one of them differentiates or undergoes anoikis. In case of a leaf, the latter refers to both daughters. Analyses of the trees confirmed that hyper-methylation of genes indeed follows repair (Figure 6A), although not every repair event results in hyper-methylation. Moreover, the clone of the affected cell overtakes the crypt in a few cases only in the course of clonal competition, i.e., not any hyper-methylation event becomes fixed. On average, only one out of *N_ISC_* events affecting ISCs leads to such a fixation, where *N_ISC_* is the average number of ISCs in the system. In all other cases, the clone carrying the hyper-methylation becomes washed out [19]. Thus, on average, N_ISC_ repair events in ISCs are required for fixation of a hyper-methylation.

Most of the genes affected by DNA hyper-methylation are transcriptionally repressed, H3K27me3 targets in controls (such as gene G2 and G4, Figure 6B). Accordingly, the expression of these genes does not change during hyper-methylation, while they lose H3K27me3 (Figure A2B). However, genes that are H3K4me3 targets in controls also can become hyper-methylated by chance, although with low frequency. This happens only if strong H3K4me3 de-methylation subsequent to cell division [21] and repair coincide in a cell. These genes become transcriptionally down-regulated during hyper-methylation, while they lose both H3K27me3 and H3K4me3 (Figure 6B). As their expression does not affect the clonal competition in the crypt, the respective ISC can win this competition with a probability of 1/*N_ISC_*.

DNA methylation measured by microarrays commonly represents an average over cells of hundreds or thousands of crypts and not over a single crypt. Thus, the switch-like changes in a single crypt appear as long term drifts as different crypts switch at different times. This effect is illustrated in Figure 6C providing the DNA methylation level of all genes averaged over all cells and ten different crypt simulations. Clear drifts are only seen for genes that become frequently hyper-methylated. Hyper-methylation of promoters of genes that are expressed at higher levels are hard to identify, although they are stably present in a number of crypts. That means, the described aging under repeated repair induces a tissue heterogeneity on the crypt level while the crypts themselves are rather homogenous due to the ongoing monoclonal conversions.

## 4. Discussion

Age-related hyper-methylation of gene promoters has been observed in ISCs. We here provided a computational model that allows the simulation of the epigenome of aging ISCs and their descendants. Applying the model, we demonstrated that drifts of the DNA methylation due to limited inheritance of histone modification states settle after a few weeks in the fast proliferating ISCs. Based on these results, we suggest that age-related promoter hyper-methylation in the intestine is a result, at least in part, of DNA repair processes. Similar conclusions have been derived examining the effects of targeted induction of DSBs in a gene promoter [43].

The basic assumption enabling the described mechanisms is a positive auto-feedback on the DNMT1 activity. This feedback potentially stabilizes un-methylated promoter DNA and, thus, high gene expression at low DNMT3a/b activity (compare Figure 3). In parallel, it provides the opportunity of DNA methylation switches between low and high DNA methylation states. For highly expressed genes, such switches require strong fluctuations in the DNA methylation level. In weakly expressed genes and particularly in H3K27me3 target genes, the required fluctuations are smaller, and these switches become more likely. These model results are consistent with experimental observations demonstrating that genes that become hyper-methylated with age are strongly enriched in H3K27me3 target genes [14].

As DNA methylation drifts due to limited inheritance of histone modifications [13], the drifts resulting from DDR represent a process of equilibration. However, in the case of repair-induced drifts, it represents an equilibration between bistable states and not just slow adaptation of a single stable state. Both drift processes require that the affected genes are initially in a low DNA methylation state. Thus, our model for the intestine applies in particular to those genes that are expressed in the embryonic tissue and become repressed in the adult tissue [40].

In the model presented, we treated DNA methylation as a deterministic process (difference equation approach). Fluctuations around a steady regulatory state arise only from fluctuations of histone modification states that change the activity of the de novo DNMTs or from changes of this activity during DNA repair processes. In CpG-rich promoters where (de-)methylation of individual CpGs does not strongly affect the average methylation level of the promoter, our approach provides an appropriate approximation. For other cases, an explicit simulation of the methylation process of individual CpGs of the promoter [13,44] might be more appropriate.

Considering a weakly methylated gene promoter, a temporary increase in the de novo DNA methylation activity enables an increase in its DNA methylation level and, if this increase is sufficiently large, pushes the gene into the attractor of the high DNA methylation state. A reversion of this switch is very unlikely: (i) as a consequence of the established DNA methylation hysteresis; and (ii) because the increased DNA methylation suppresses recruitment of H3K4me3 and thus suppresses a decrease of the de novo methylation activity.

DNA methylation changes of individual CpGs are acquired rather stochastically [45]. Accordingly, one can expect even switches between coexisting DNA methylation states to occur also without changing the de novo methylation activity. This would allow for repair-independent age-related drifts until a dynamic equilibrium between the coexisting states is reached. Thus, as mentioned above, the described drift is one potential drift among others. It might be particularly effective in repair-deficient tissue where this deficiency induces collateral DNA damage. This fits with the idea that chemotherapy induced DNA damage supports the CpG-Island Methylator Phenotype (CIMP) [46]. Fast drifts can be expected also under high-fat diet known to increase the frequency of strand breaks [47].

In our model, the mechanism underlying methylation changes during DDR rests on a release of KDM2b from the affected gene. As mentioned, such a mechanism has been described recently, following DSB formation [32]. As KDM2b has multiple functions, this release affects at least the H3K4me3 (de-) modification balance [48] and the protection of the promoter from DNA methylation [34]. These two mechanisms are antagonistic, as increased H3K4me3 represses recruitment of de novo DNMTs [27], and increased DNA methylation represses recruitment of H3K4me3 HMTs [28]. Thus, one can speculate whether increased de novo DNA methylation serves to reduce the risk of unrequested gene activation following increased H3K4me3 HMT activity.

Assuming a realistic number of about 50 DSBs per cell and day (cell cycle time in mice ISCs) [49], less than 20,000 DSBs occur per cell and year. A successful fixation of a hyper-methylation event requires on average *N_ISC_* repair events in ISCs, i.e., each ISC has to be hit on average once. Thus, even if every DSBs would affect, on average, one of the (>20,000) genes, far more than a year would be required to hyper-methylate the susceptible genes within the cells of a mouse crypt, as not every repair event leads to hyper-methylation. Accordingly, the described process can be considered to proceed on a life-long time scale.

The assumption of one repair per 100 genes and cell cycles relates to about 200 DSBs per 20,000 genes and cell cycles. This frequency was sufficient to observe fixation of hyper-methylation events of single genes in a crypt within 300 simulated days. However, this required that the de novo DNA methylation probability is sufficiently increased during repair. We found that a weaker repair effect can hardly be compensated by higher numbers of DSBs. In our simulations, we assume that successful DNA repair does not result in apoptosis and does not reduce the competitive potential of the cells. Otherwise, the described drifts would be slowed down.

Besides providing a potential explanation for DNA hyper-methylation in the aging intestine, our model does also provide first insights into the link between tissue specific proliferation history and epigenetic profiles of cells. Without any assumption about a specific gene regulation during lineage specification, our model shows developing differences during this process on top of the transcriptional regulation. Thus, changes of the proliferation scenario within the intestine can be expected to lead to aberrant epigenetic regulation. Our model also enables the simulation of loss and gain of function manipulations of epigenetic modifiers, as well as of changes of the Wnt- and Notch-pathway activity relevant for proliferation. Thus, we expect future simulation studies to contribute to a better understanding of aberrant DNA methylation during early steps of tumor formation.

## Abbreviations

ISC	Intestinal stem cell
PC	Paneth cell
GC	Goblet cell
EC	Enterocyte
DSB	Double-strand break
DDR	DNA damage repair
HDM	Histone demethylases
HMT	Histone methyltransferase
DNMT	DNA methyltransferase

## Appendix A. Model Description

### Appendix A1. Model of the Epigenetic Regulation

The binding probabilities of the HMTs for H3K4me3 and H3K27me3, *θ^K^*, are calculated assuming a positive feedback between the histone mark *K* (*K* = 4, 27) and the recruitment of the respective HMTs [21]. They are given by:

(A1) $$\theta^{K}=1/\left(1+\exp\left(\varepsilon_{0}^{K}+\varepsilon_{BS}^{K}\left(1-m_{CpG}\right)+\varepsilon_{HM}^{K}N_{H}m_{K}+\lambda^{K}\log\left(T\right)\right)\right)$$

where $\varepsilon_{0}^{K}$ is the ground enthalpy per bound HMT complex and $\varepsilon_{BS}^{K}$ and $\varepsilon_{\mathrm{HM}}^{K}$ are the free energies of HMT binding to DNA and to histone mark *K*, respectively. They are all scaled by the Boltzmann unit. The factor $\lambda^{K}$ is (−1) for H3K4me3 and (+1) for H3K27me3.

Histone modification dynamics is simulated as a stochastic process with the following modification (*r_mod_*) and de-modification (*r_de_*) probabilities for unmodified and modified nucleosomes, respectively [50]:

(A2) $$r_{mod}=C_{mod}^{K}\theta^{K},\;r_{de}=C_{de}^{K}$$

Here, $C_{de}^{K}$ and $C_{mod}^{K}$ are the de-modification and modification constant of mark K, respectively. The modification state is updated every 10th time step (time step: Δ*t* = 20 s). The DNA methylation level *m_CpG_* is updated after each cell division according to Equation (3). All parameters of the epigenetic machinery are specified in Table A1.

**Table A1:** Parameter set of the epigenetic machinery. * scaled by the Boltzmann unit. Parameter values in brackets are those applied during repair.

Parameter	Value	Description
	Histone modification	
$\varepsilon_{0}^{4}$*,* $\varepsilon_{0}^{27}$	10.0, 11.0	ground enthalpy per bound HMT *
$\varepsilon_{BS}^{4}$*,* $\varepsilon_{BS}^{27}$	−5.0, −5.0	free energy of CpG binding *
$\varepsilon_{HM}^{4}$*,* $\varepsilon_{HM}^{27}$	−0.8, −0.8	free energy of histone binding *
$C_{mod}^{4}$*,* $C_{mod}^{27}$	0.1, 0.1	modification constant
$C_{de}^{4}$*,* $C_{de}^{27}$	0.01 (0), 0.01	de-modification constant
*N_H_*	10	number of cooperative nucleosomes
	DNA methylation	
*D_main,0_*	0.99	probability of maintaining DNA methylation
*D_novo,0_*	0.1 (0.3)	probability of de novo DNA methylation
$E_{CpG}^{4}$*,* $E_{CpG}^{27}$	6, 4	interaction energy between HMTs and DNMTs *
*E_m,0_*	2	ground enthalpy per bound maintenance DNMT *
*E_m,1_*	−10	free energy of methyl-CG binding *

### Appendix A2. Model of Transcriptional Regulation

We assumed that the transcription of the genes depends on the modification levels *m_4_* and *m_27_* of their promoters and on the TF network [21]. It is updated in a self-consistent way every 10th time step for each gene *i* considering the previous changes of the histone modification states of all genes. The changes of the transcription per update are given by:

$$\Delta T_{i}=\frac{P_{max}\left(m_{4,0}+m_{4}\right)\left(1-m_{\mathrm{27}}\right)}{F_{i}^{TF}+\left(P_{max}-1\right)}F_{i}^{TF}-\delta T_{i}$$

where *P_max_* is the maximum promoter activity and *δ* the transcript degradation constant. The constant *m_4,0_* = 0.1 ensures that genes can be transcribed with a small rate even if the promoter is devoid of H3K4me3 (*m_4_* = 0). $F_{i}^{TF}$ is the regulation factor for gene *i* representing impact of the TF-network [31]. It is given by:

$$F_{i}^{TF}=\underset{j}{\prod }\frac{1+T_{j}\exp\left(\varepsilon_{i,j}^{A}\right)}{1+T_{j}}$$

where the product runs over all TFs regulating gene *i* and $\varepsilon_{i,j}^{A}$ is the energy change of polymerase binding to the promoter of gene *i* in presence of the TF *j*. Details about the underlying regulatory principles, which are based on thermodynamics can be found in [31]. The parameters of the transcriptional machinery are summarized in Table A2.

**Table A2:** Parameter set of the transcriptional machinery. In the simulations, a genome assembled from 100 genes is applied. * scaled by the Boltzmann unit.

Parameter	Value	Description
*P_max_*	100	maximum promoter activity
*δ*	0.1	transcript degradation constant
*ε_A_*	1/−1	free energy change of polymerase binding for activators/repressors *
*L_reg_*	2.000 bp	length of the regulatory region
*L_pro_*	5 bp	length of the promoter region
*L_cod_*	7 bp	length of the coding region

### Appendix A3. Crypt Model

Our 3D computational model of the small intestinal crypt has been described previously [19,20]. It describes cells of the epithelium as individual objects that move on a basal membrane of fixed shape. Thereby, they from contacts to other cells, can specify, grow, and divide. They undergo anoikis and are removed from the system if they lose the basal membrane contact. Fate decisions of the cells are assumed to be regulated by extrinsic stimulation of Wnt- and Notch- signaling. ISCs are characterized by high Wnt- and Notch-signaling. The model considers specification of these cells into three lineages: enterocytes (ECs), Paneth cells (PCs), and Goblet cells (GCs). ISC specify into ECs or PCs if the Wnt-signaling or the Notch-signaling fall below a threshold value, respectively. In cases where both signals fall below a threshold, the ISCs adopt (via the EC or PC fate) the GC fate. The Wnt-activity depends on the cell’s position P along the crypt-villus axis. It decreases with increasing distance from the bottom of the crypt (Figure 2A). Accordingly, ISCs that move up the crypt axis decrease Wnt- signaling and specify into absorptive ECs at a certain position P_1_. The Notch-activity is controlled by the cell’s neighbors (Figure 2B). Secretory cells (PCs, GCs) activate Notch-signaling in neighboring cells via ligand-receptor interactions. Accordingly, ISCs (ECs) that lose PC- (GC-) contacts decrease Notch-signaling and specify into secretory PCs (GCs) if the number of such neighbors, N, falls below a threshold *N_1_* (*N_2_*). In similar ways, PCs that lose Wnt-signaling (*P* > *P_1_*) specify into GCs. All fate decisions are assumed to be reversible as long as the cells are not terminally differentiated. ECs and GCs become terminally differentiated if the Wnt-signaling falls below a second threshold value at position *P_2_* > *P_1_*. PCs become terminally differentiated under high Wnt-activity in cases where they have finished a last cell cycle subsequent to specification. As long as they are not terminally differentiated, all cells are capable of proliferating. Proliferating cells increase their volume with a defined growth rate. If their volume reaches twice the initial volume *V_0_* they divide into two cells. Their daughters can start proliferation again if they are not subject to contact inhibition. Cells are considered to be contact inhibited if their volume is smaller than a threshold volume *V_p_*, e.g., due to a compression induced by their neighbors. Cells try to relax such compression by changes of their position or radius. In other words, ongoing proliferation generates changes in adhesion, deformation, and compression forces which drive cell movement. All specified cells actively generate additional forces such that ECs and GCs perform an effective movement up the crypt-villus axis and PCs down this axis. Cells that leave the crypt (*P* > 30) are removed from the crypt. The parameter set used in our simulations is given in the Table A3.

**Table A3:** Parameter set of the crypt model [19].

Symbol	Value	Parameter	References
**Parameter of the cell model**			
*V_0_*	4/3π (5 µm)^3^	minimal volume of an isolated cell	estimated
*τ*	14 h	cell growth time	results in an effective cell cycle time ~24 h
*E*	1 kPa	Young modulus	[51]
*ν*	1/3	Poisson ratio	
*ε_c_*	200 µN/m	cell-cell anchorage	
*V_p_*	0.88 V_0_	threshold volume for contact inhibition	set
**Parameter of the Basal Membrane model**			
*z_0_*	150 µm	length of the crypt	set, according to properties of the crypt shape [20]
*r_0_*	60 µm	crypt radius at the crypt-villus junction	
*λ_1_*	0.25	shape parameter 1	
*λ_2_*	0.1	shape parameter 2	
*Ω*	0.95	optimal cell-BM distance in cell radii	set
$\varepsilon_{K}^{Paneth}$	35 10^−12^ Nm	maximum cell-knot interaction energy of PCs	set
$\varepsilon_{K}^{other}$	5.5 10^−12^ Nm	maximum cell-knot interaction energy of all other cells	ensuring apoptosis rates < 5% [52]
**Parameter of crypt dynamics**			
*η_c_*	5 10^10^ Ns/m^3^	friction constant for cell-cell friction	[51]
*η_BM_*	3.2 Ns/m	friction coefficient for cell-BM friction	fit: turnover
*η_VO_*	400 Ns/m	friction coefficient regarding volume changes	[51]
$F_{A}^{Paneth}$	7.5 nN	active migration force of PCs	fit: distribution of PCs
$F_{A}^{other}$	4.5 nN	active migration force of all other cells	fit: turnover and Brdu data
**Parameter of the lineage specification and differentiation model**			
*P_1_*	−129 µm	position of the Wnt- threshold TP_Wnt_ for priming	fit: size of the PC compartment
*P_2_*	−87.5 µm	position of the Wnt- threshold TD_Wnt_ for differentiation	fit: turnover and Brdu data
*N_1_*	3	minimum number of Notch-ligand expressing neighbors in the niche	set: refers to TD_Notch_ /LP^Paneth^ and TD_Notch_/LP^Goblet^ [20]
*N_2_*	1	minimum number of Notch-ligand expressing neighbors outside the niche	
*τ* *^P^*	8 weeks	average PC lifespan with contact to SCs	[19]
τ^A^	12 h	max. PC lifespan without contact to SCs	

## Appendix B. Additional Simulation Results

### Appendix B1. Bistable DNA Methylation States

For functional tissue, a high precision of the maintenance of DNA methylation is reported, paralleled by a low de novo methylation per cell cycle [53]. Thus, we set the parameters *D_main,0_* and *D_novo,0_* to 0.99 and 0.1, respectively. The two additional parameters, required to enable bistable DNA methylation states, currently cannot be derived from experiments. E_m,0_ describes the energy that has to be overcome by maintenance DNMTs to bind to DNA, while E_m,1_ describes how this energy is lowered if the CpGs located within the promoter are methylated (both scaled by the Boltzmann unit). In our model, only a weak energy barrier has to be overcome *E_m,_*_0_ = 1–3 and a fully methylated region enables strong binding: *E_m,0_* < *E_m,0_* ~ 10. Figure A1 shows solutions for different parameters. Increasing *E_m,0_* extends the bistable region and, thus, will suppress the hyper-methylation scenario. Similar effects result from a decrease of *E_m,1_*. Thus, changes, for example, of the concentration of MBD4 will affect the hyper-methylation scenario.

### Appendix B2. Simulation of Aging

The parameters of the epigenetic regulation model have been chosen to reproduce experimental findings on epigenetic state distributions. Most promoters are H3K4me3 modified, fewer are H3K27me3 modified, while only a small portion is bivalent. Figure A2A provides the full regulatory state information (*T*, *m_4_*, *m_27_*, *m_CpG_*) for all genes as observed during damage-free aging. Figure A2B shows the differences between the regulatory states during damage-free and repair-induced aging.

The parameters of the repair model have been chosen to demonstrate repair-induced epigenetic drifts within a time window of 300 days, which remains computationally tractable. Single hyper-methylation fixation events are already seen in this time window for a repair frequency of one repair per cell cycle and cell. For two repair events per cell cycle and cell, we observed on average one hyper-methylation fixation event per crypt. Figure A3 provides insights into the dependence of DNA hyper-methylation on the frequency of repair as well as on the dependence of the assumed change of D_novo,0_ during repair.
